# Genetics and Genomics of Infectious Diseases in Key Aquaculture Species

**DOI:** 10.3390/biology13010029

**Published:** 2024-01-04

**Authors:** Nguyen Hong Nguyen

**Affiliations:** School of Science, Technology and Engineering, University of the Sunshine Coast, Maroochydore, QLD 4558, Australia; nnguyen@usc.edu.au

**Keywords:** genetic basis, genomic architecture, disease tolerance, population resilience, climate change

## Abstract

**Simple Summary:**

Diseases pose a significant challenge for aquaculture, exacerbated by changing weather conditions. The sector has explored various strategies, including maintaining a clean environment and employing vaccines, to combat these diseases. However, these solutions are effective only against specific diseases and species. In our recent research, we investigated the use of genetics to enhance disease resistance in three crucial species: white leg shrimp, striped catfish, and yellowtail kingfish. Our findings indicate that the studied populations of these species possess genes that can be inherited, imparting greater resistance to diseases such as White Spot Syndrome Virus, Bacterial Necrotic Pancreatitis, and skin fluke. By selectively breeding animals with these resistant genes, we successfully increased resistance within the population, promoting overall fish health and boosting production. Additionally, we examined these genes and utilized computer models to predict the most resistant individuals for breeding to combat diseases. Looking ahead, our focus is on omics technologies, precision farming systems, and advanced algorithms to further enhance the disease resistance of these species, thereby making aquaculture more sustainable and resilient against threats.

**Abstract:**

Diseases pose a significant and pressing concern for the sustainable development of the aquaculture sector, particularly as their impact continues to grow due to climatic shifts such as rising water temperatures. While various approaches, ranging from biosecurity measures to vaccines, have been devised to combat infectious diseases, their efficacy is disease and species specific and contingent upon a multitude of factors. The fields of genetics and genomics offer effective tools to control and prevent disease outbreaks in aquatic animal species. In this study, we present the key findings from our recent research, focusing on the genetic resistance to three specific diseases: White Spot Syndrome Virus (WSSV) in white shrimp, Bacterial Necrotic Pancreatitis (BNP) in striped catfish, and skin fluke (a parasitic ailment) in yellowtail kingfish. Our investigations reveal that all three species possess substantial heritable genetic components for disease-resistant traits, indicating their potential responsiveness to artificial selection in genetic improvement programs tailored to combat these diseases. Also, we observed a high genetic association between disease traits and survival rates. Through selective breeding aimed at enhancing resistance to these pathogens, we achieved substantial genetic gains, averaging 10% per generation. These selection programs also contributed positively to the overall production performance and productivity of these species. Although the effects of selection on immunological traits or immune responses were not significant in white shrimp, they yielded favorable results in striped catfish. Furthermore, our genomic analyses, including shallow genome sequencing of pedigreed populations, enriched our understanding of the genomic architecture underlying disease resistance traits. These traits are primarily governed by a polygenic nature, with numerous genes or genetic variants, each with small effects. Leveraging a range of advanced statistical methods, from mixed models to machine and deep learning, we developed prediction models that demonstrated moderate-to-high levels of accuracy in forecasting these disease-related traits. In addition to genomics, our RNA-seq experiments identified several genes that undergo upregulation in response to infection or viral loads within the populations. Preliminary microbiome data, while offering limited predictive accuracy for disease traits in one of our studied species, underscore the potential for combining such data with genome sequence information to enhance predictive power for disease traits in our populations. Lastly, this paper briefly discusses the roles of precision agriculture systems and AI algorithms and outlines the path for future research to expedite the development of disease-resistant genetic lines tailored to our target species. In conclusion, our study underscores the critical role of genetics and genomics in fortifying the aquaculture sector against the threats posed by diseases, paving the way for more sustainable and resilient aquaculture development.

## 1. Introduction

Infectious diseases cause substantial economic losses in the aquaculture sector [1]. Estimating the exact figure with high accuracy is challenging; for example, an estimated annual revenue loss globally because of viral diseases is approximately USD 3–5 billion for the shrimp sector alone [2]. Predictions suggest that the impact of disease outbreaks on the aquaculture sector will become more severe due to the unprecedented effects of changing environments [3,4]. Apparently, climate change can exacerbate the impact of diseases on animal health by altering the behavior of pathogens, hosts, disease vectors, transmission rates, or the distribution of competitors, predators, and parasites within ecosystems [5]. Elevated water temperatures, for instance, can accelerate the development of highly infectious pathogens, potentially leading to disease outbreaks with severe financial repercussions [6].

Controlling diseases in aquaculture is crucial to ensure healthy animals and maximize yields. Common methods and strategies used to manage and control diseases in agriculture include biosecurity measures, sanitation, quarantine, chemical treatments, and therapeutic approaches such as diets, probiotics, or improved farming practices like crop rotation and vaccines [7]. Sanitation involves cleaning and disinfecting to prevent the transfer of pathogens. Quarantine measures are essential to prevent the introduction of new diseases and protect pathogen-free farms. Chemical treatments, such as antibiotics, have raised concerns due to their potential impacts on human health and the natural environment, as well as the development of resistance [8].

While vaccines have shown promise in controlling diseases in aquaculture species, their effectiveness can vary depending on several factors, including disease and species specificity [9,10]. There is a growing interest in controlling infectious diseases in an environmentally sustainable manner. Integrated farming systems, such as those involving fish, shrimp, and aquatic plants, or crop plants like rice, as well as the rotation of these species, can disrupt the life cycle of many disease-causing pathogens and reduce their build-up in aquaculture farms [11]. However, these measures are often temporary and may not always be cost effective and sustainable in the long term.

Genetics and genomics hold promise for providing sustainable solutions through the development of disease-resistant strains [12,13]. Specifically, quantitative genetic theory [14] provides the framework for systematically improving disease resistance in host populations, both through selective breeding and, more recently, through the integration of genomic data [15,16]. This approach has been successfully applied in various fields, including agriculture and animal breeding, to enhance disease resilience in animal and plant populations [17]. Conventional genetic improvement programs combine phenotypic measurements with pedigree information. Phenotypic measurements involve measuring traits such as disease incidence, severity, or pathogen load in host populations [18]. A pedigree is a family tree that keeps records over multiple generations and is maintained through physical tagging methods, such as PIT tags for fish and visible elastomer tags for crustaceans, or DNA markers. Analysis of pedigree and phenotypic information is often conducted to obtain genetic parameters (heritability, correlations), providing primary inputs for genetic improvement programs and estimating individual genetic merits, known as estimated breeding values (EBVs) for selection. Across species, heritability for disease resistance to various pathogens (bacteria, viruses, parasites, or other pathogens) is moderate to high (h^2^ = 0.09–0.41), indicating that genetic factors play a significant role in determining the trait and it can respond to artificial selection [19,20]. In addition to population parameters, genetic evaluation of pedigree and disease phenotypes is conducted to estimate EBVs for all individuals in the pedigree [21]. Based on these EBVs, individuals with higher genetic resistance to the disease are selected as parents for the next generation. This approach has achieved significant genetic gain, ranging from 4% to 15% for economically important traits including disease resistance to various pathogens in fish, crustaceans, and mollusks [22,23]. Despite these successes, genetic improvement programs for aquaculture species often focus solely on improving resistance to a specific pathogen or selecting a single trait. Breeding objectives should be broadened by incorporating disease resistance alongside commercial traits like growth rate, meat quality, and reproductive performance. Furthermore, infectious disease resistance can be influenced by environmental factors, making it essential to evaluate resistance under different environmental conditions relevant to the target population.

Recently, omics technologies, when integrated with pedigree and phenotypes, have enhanced our understanding of the genetic basis of infectious diseases, guided the development of novel therapeutics and vaccines, and enabled more precise and personalized approaches to disease management and prevention [24]. Specifically, genomics involves the study of an organism’s entire genome, including its genes and non-coding sequences. Sequencing the genomes of infectious agents (e.g., bacteria, viruses, parasites) helped us to understand their genetic diversity, transmission pathways, evolution, and disease dynamics [25]. More importantly, genome sequencing of aquaculture species helped us to understand the genetic factors in the host that influence susceptibility, resistance, and immune response to infectious diseases. In this context, genome sequencing also enables the selection of individuals based on their DNA information. This approach is known as genomic selection [26], which involves using genome-wide markers or sequencing data to predict their risks of diseases and their genomic breeding values for desirable traits, such as disease resistance. Studies in aquaculture species employing various algorithms, from mixed models to machine or deep learning, have shown that recent high-throughput genome sequencing platforms can provide genomic information to achieve moderate-to-high levels of prediction accuracy for disease traits [27,28]. After the prediction model is trained, breeders can apply it to individuals whose disease resistance is unknown, such as young animals. There are several benefits of genome-based selection for disease resistance, mainly including early identification and selection of disease-resistant individuals to reduce the generation time, increase accuracy to achieve faster improvements, reduce the cost of data recording, and improve genetic diversity [29]. In short, genome-based selection is a powerful tool for enhancing disease resistance of aquaculture species, contributing to sustainable agriculture and improved animal health.

There are also other omics techniques [30]. Transcriptomics studies differential gene expression between individuals or groups with different disease outcomes to discover genetic factors that contribute to resistance or susceptibility or identify genes that are upregulated or downregulated in response to infection. Proteomics assists in identifying host proteins or protein signatures that can serve as biomarkers for disease diagnosis, prognosis, or monitoring. Metagenomics [31] studies the composition and functional potential of microbiomes to understand their role in infectious diseases and identify ways to manipulate the microbiome for therapeutic purposes [32]. Functional genomics involves manipulating genes to understand their functions. This can include techniques like CRISPR-Cas9 gene editing to study the impact of specific genes on disease susceptibility or pathogen virulence [33,34].

This study is not intended to offer an exhaustive review of the existing literature on these subjects. A more comprehensive exploration of these themes can be found in recent review articles [35,36]. The current paper aims to emphasize the key discoveries derived from our research employing genetic and genomics methodologies to enhance disease resistance, focusing on three principal aquaculture species: white leg shrimp (*Litopenaeus vannamei*), striped catfish (*Pangasianodon hypophthalmus*), and yellowtail kingfish (*Seriola lalandi*). Furthermore, this discussion encompasses emerging technologies, including precision agriculture systems and AI algorithms, and future research directions aimed at expediting genetic enhancements for our targeted species.

## 2. Genetic Factors in Common Diseases of White Shrimp, Striped Catfish, and Yellowtail Kingfish

### 2.1. Genetic Susceptibility

Our studies investigated the heritable genetic components of susceptibility or resistance to viral, bacterial, and parasitic diseases in three species: white leg shrimp, striped catfish, and yellowtail kingfish (Table 1). To unravel the genetic basis of these traits, we developed standardized challenge test protocols for individual species, conducted field tests, and meticulously recorded disease resistance data [37,38]. The disease data were collected in a form of binary expression (absence of the studied disease = 0 and presence = 1) or as continuous variables, representing the time (in days) from the challenge test to mortality.

For analysis, we employed various statistical models. Continuous data were subjected to a linear mixed model (LMM), while binary data were analyzed using a generalized linear mixed model (GLMM). The GLMM, also known as the threshold model, assumes that multifactorial diseases result from an underlying continuous character, known as liability. The liability heritability is subsequently transformed back to the original observed (0/1) scale, and the h^2^ estimates on both liability and observed scales are generally of similar magnitude [37,38]. Importantly, we observed high genetic correlations of estimated breeding values (EBVs) between LMM and GLMM models, suggesting their interchangeability for practical genetic evaluation programs aimed at enhancing disease resistance in aquaculture species.

Furthermore, our investigation revealed that the heritability of disease resistance traits, such as resistance to White Spot Syndrome Virus (WSSV), varied with the duration of the challenge test. For instance, the heritability (h^2^) decreased from 0.38 to 0.23 in the initial stages of infection to a range of 0.01 to 0.14 in the later phases of the challenge test experiment [38]. This reduction in heritability can be attributed to the fact that shrimp WSSV resistance relies more on innate immune responses during the early infection stages. Towards the end of the challenge test, shrimp resistance appeared to be increasingly influenced by non-genetic factors, including environmental conditions and stress induced by high viral density.

Our findings align with earlier studies conducted in laboratory and farm settings [19,20]. The literature has generally reported low heritability estimates for WSSV resistance in larger, grow-out-size shrimp (around 10%) [42]. Similar results were obtained for skin fluke disease in yellowtail kingfish [40,41]. Cumulatively, our findings, along with those from the existing literature, underscore the presence of a heritable genetic component for disease resistance. Selective breeding for improved resistance has been proved as an effective strategy for mitigating infectious diseases in aquaculture species.

### 2.2. Genetic Correlations of Disease Resistance with Complex Traits

To elucidate the genetic interplay between disease resistance and economically significant traits, we carried on estimating their genetic correlations (Table 2). Within both shrimp and striped catfish populations, we obtained favorable genetic correlations (ranging from 0.44 to 0.86 ± 0.03 to 0.10) between disease resistance and survival traits [37]. These findings suggest that selecting for enhanced resistance against *E. ictaluri* or WSSV could have advantageous effects on survival traits, both during hapa rearing prior to bacterial challenge and throughout communal grow-out, from tagging to harvest.

However, the genetic association of these diseases with growth traits was less conclusive. This uncertainty is evident from the substantial standard errors in the genetic correlation estimates between these two trait categories within our populations. In the existing literature, genetic correlations between body weight and disease resistance have been reported as either positive or non-significant [19]. Nevertheless, some studies have also documented negative, antagonistic genetic associations between growth and disease resistance. For instance, such correlations ranged from −0.01 to −0.33 in rainbow trout [43] and from −0.54 to −0.66 in Pacific white leg shrimp [44]. Recent studies have even shown weak or non-significant genetic relationships between these two traits in rainbow trout [45] and banana shrimp [46]. In a meta-analysis conducted by Nguyen (2021) [19], the weighted mean genetic correlation (*r_g_*) between weight and disease resistance to different pathogens did not reach significance (*r_g_* = 0.13, 95% CI = −0.07–0.33).

In summary, our findings highlight the need for further research and data accumulation to re-evaluate and confirm the genetic correlations between harvest weight and disease resistance in these populations.

## 3. Selection to Enhance Disease Resistance

### 3.1. Genetic Gains

Possessing the heritable genetic variation in disease resistance within these populations, we initiated selection programs aimed at enhancing resistance. The results were remarkable, with a substantial genetic gain averaging 12.9% per generation [23]. The significant genetic improvement in the animals’ resistance capacity against various diseases, including bacteria, viruses, and parasites, clearly demonstrates the success of our selective breeding program in developing disease-resistant lines. These lines exhibited significantly higher survival rates than the control group under commercial conditions.

The genetic progress achieved for these traits within these populations stems from the extensive genetic variability within our selected population, which was created from a synthetic population comprising genetically divergent lines [37,47]. The magnitude of the genetic changes in our population aligns with findings from previous studies in aquatic species, ranging from 6% to 18% per generation [21,48].

Another factor contributing to the success of our genetic programs was our commitment to standardizing the experimental procedures, from early rearing to the challenge tests. This encompassed control over the microenvironment in the experimental tanks and the sampling frequency. Additionally, we accounted for all potential environmental effects, including the age of the animals during the challenge test. We applied a combination of between- and within-family selection to choose breeding candidates for future generations, deliberately avoiding closely related siblings to minimize inbreeding and maintain genetic diversity in these closed nuclei populations. The production of many experimental families (averaging 150 full and half-sib families per generation) and multiple individuals per family provided ample pedigree and phenotypic data for genetic evaluation, thus enabling us to achieve a high rate of genetic improvement for disease resistance traits. Nevertheless, future studies should assess the effects of genotype–environment interaction and the co-effects of other pathogens in the selected populations of yellowtail kingfish, white leg shrimp, and striped catfish.

We recommend that breeding programs should undergo regular assessments to monitor the genetic progress of the selection population and to refine breeding technologies. In our breeding programs aimed at improving disease resistance in three species showcased here, a control group was recreated for each generation based on the population mean. This control group served as a benchmark for estimating genetic gains by comparing the estimated breeding values of the selection line and the control group within the same spawning season or generation, or by evaluating the progeny of the selection line across successive spawning seasons. Our estimation of genetic gains using mixed model procedures relies on the presence of genetic connectedness between generations, which enables the separation of genetic effects from environmental factors, thus minimizing bias.

### 3.2. Effects of Selection for Enhanced Disease Resistance on Commercial Traits

#### 3.2.1. Effects on Survival and Growth

The effects of selection for improved resistance on growth traits were formally assessed exclusively in white leg shrimp. Intriguingly, our genetic programs to enhance WSSV resistance yielded favorable outcomes for both survival and growth traits, as documented by Trang et al. (2019) [23]. The high-WSSV-resistance families displayed a notable 7% increase in average body weight compared to the low-resistance family group. Furthermore, the survival rate among high-WSSV-resistance shrimp exceeded that of the control group by 17%. These findings were substantiated by a positive genetic correlation observed between the WSSV-resistance and growth traits, albeit with modest estimates (*r_g_* = 0.07). However, based on the weak genetic correlation estimates of body weight with fluke disease in YTK [49] and with BNP disease in striped catfish [37], correlated responses in growth traits may not be statistically significant. To ascertain these effects in future breeding programs for these species, accumulation of more data in future generations is needed for further analysis. To date, there is still limited published information on correlated genetic changes in commercial traits as a direct consequence of selection for improved disease resistance in other species. This underscores the need for further comprehensive studies in this area to enhance our understanding of how to effectively manage genetic responses within artificial selection programs for aquatic animal species. Continued research in this domain is essential for advancing our ability to optimize genetic selection in aquaculture and related fields.

#### 3.2.2. Effects on Immune Response in Shrimp

In our study [50], we explored genetic alterations in four critical immunological parameters—total hemocyte count (THC), phenoloxidase (PO), superoxide dismutase (SOD), and lysozyme—within a white leg shrimp population that had been selectively bred for high and low resistance to White Spot Syndrome Virus (WSSV). This study was initiated by the pivotal role played by the innate immune system in providing non-specific defenses against external pathogens in crustacean species. To accomplish this, we collected hemolymph samples from 38 shrimp families, each averaging around 24 g in weight. These samples were subsequently subjected to analysis to assess THC, PO, SOD, and lysozyme activities. Our analysis yielded results indicating that there were no significant disparities in the activities of the tested immune parameters between the groups characterized by high and low WSSV resistance (Table 3). Additionally, when scrutinizing phenotypic correlations between immune parameters and traits related to growth or disease resistance, we found that, with one exception, these correlations were not statistically significant. Specifically, we observed a moderate negative correlation estimate between PO and WSSV resistance (−0.405). The outcomes derived from this study collectively suggest that the immune response within this white leg shrimp population remained largely unaltered following a single generation of selective breeding for WSSV resistance. As of the present, there are no published data regarding the immune response to selection for enhanced disease resistance in any penaeid species. However, research in fish [51] has also reported a lack of significant associations between these immune parameters and slaughter weight or survival rates.

#### 3.2.3. Effects on Immunological Parameters in Striped Catfish

In stark contrast, our investigation in striped catfish [52] revealed compelling evidence of significant differences in the immune responses between families selected for resistance (RF) and those susceptible (SF) to *Edwardsiella ictaluri*, the causative agent of bacillary necrosis of Pangasius (BNP disease). Within the RF group, we observed a more robust non-specific immune response compared to in the SF group. In particular, red blood cells in the RF group exhibited a gradual and delayed destruction, while white blood cell counts in this group showed a substantial increase starting at 48 h post infection (hpi), in contrast to the SF group. Furthermore, we observed that the development of monocytes and neutrophils (NEU) in the RF families outpaced that of the SF group, and the number of melano-macrophage centers (MMCs) in the RF families was notably higher than that observed in the SF group at 24–48 hpi. Beyond these observations, the specific immune response, as measured by antibody titers (ABT), was significantly higher in the RF group when compared to the SF group. Additionally, our study showed that three main immunological parameters (NEU, ABT, and MMCs), measured during the 24–48 h post-infection stage, can serve as effective indicators for distinguishing resistant from susceptible individuals. The determination model, gaining a high AUC value of 0.95, indicates its remarkable accuracy in identifying resistant and susceptible individuals within the striped catfish population.

## 4. Alternative Selection Criteria to Enhance Disease Resistance

Genetic selection aimed at enhancing disease resistance remains a formidable challenge in aquaculture species, primarily due to the involvement of pathogen challenge tests that are both costly and time consuming. Recent research endeavors [46] have explored alternative selection criteria, such as viral titer or viral load, as means to develop disease-resistant lines within white shrimp species. Although heritable genetic components for viral titers related to infectious diseases, specifically hepatopancreatic parvovirus (HPV) in banana shrimp [46] and White Spot Syndrome Virus (WSSV) in *L. vannamei* [38], have been identified, accurately measuring this trait requires examination of the hepatopancreas in sacrificed breeding candidates. Furthermore, while there is a positive genetic association between viral titer and WSSV resistance, this correlation significantly deviates from unity, implying that these are distinct traits and that selecting for reduced viral titer cannot encapsulate the full spectrum of genetic expression associated with WSSV resistance, as observed by Trang et al. in 2019 [23]. Given these challenges, there has been a growing interest in leveraging immunological parameters within selective breeding programs to bolster disease resistance across aquaculture species. In our research, we sought to acquire initial genetic parameters for immunological traits in our target species. Due to the limited sample sizes used for analyzing these immunological parameters, heritability estimates for these traits are not published due to their low reliability. Nevertheless, studies in other species have provided evidence of genetic variability in immune responses, with heritability (h^2^) estimates ranging from 0.2 to 0.3 for lysozyme in rainbow trout and h^2^ estimates of 0.16 to 0.20 for antibody titers in Atlantic salmon [53]. Furthermore, in various fish species, positive correlations have been established between disease resistance and non-specific immune factors, such as the correlation between *Aeromonas hydrophila* resistance and immunity traits in bighead catfish (*r_g_* = 0.05–0.27) [54] or *Vibrio anguillarum* and *A. salmonicida* resistance and specific antibodies in rainbow trout [55]. Also note that the correlation of resistance against *A. hydrophila* with different immunological factors exhibited both negative and positive associations (*r_g_* = −0.48 to 0.51) [56]. The findings from these studies collectively suggest that immunological parameters hold promise as indirect selection criteria within selective breeding programs, ultimately enhancing overall disease resistance in aquaculture species, as highlighted by Van Sang et al. in 2023 [52]. Currently, we are expanding our sample size to ensure a reliable estimation of genetic parameters within the populations of white leg shrimp and striped catfish under investigation. 

## 5. Genetic Variants for Disease Resistance

Traditional quantitative genetic selection has delivered a spectacular response, averaging 12.9% per generation, in bolstering resistance to different pathogens [23]. Nevertheless, this conventional approach relies on challenge tests, which present significant practical challenges, including concerns related to biosecurity, labor intensiveness, time consumption, and high costs. Considering these obstacles, we have identified molecular genetic markers that can facilitate gene- or marker-assisted selection for traits that are inherently challenging and costly to measure, such as disease resistance. To advance in this direction, we conducted restricted-sites-associated DNA sequencing (RAD-seq) on a cohort consisting of 752 yellowtail kingfish (YTK) and 560 striped catfish individuals.

**Figure 1:**
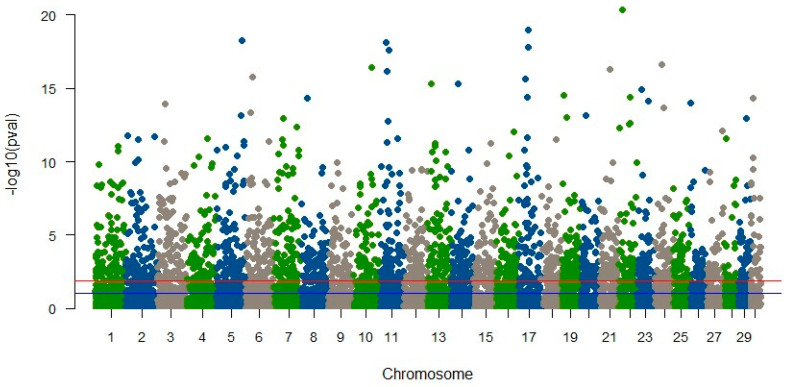
Manhattan plot of significant SNPs associated with BNP disease in striped catfish (Vu et al., unpublished results). The horizontal blue and red lines indicate significant probability greater than 5% and 1%, respectively.

In YTK, our investigation yielded no markers associated with the animals’ susceptibility to skin flukes [57]. This outcome may be attributed to multiple factors, including the limited representation of diseased fish in our study (only 4%), the utilization of shallow sequencing strategies, potential recombination during the course of line development, or insufficient linkage disequilibrium (LD) between markers and the genes responsible for disease susceptibility [49]. In contrast, our analysis of striped catfish identified numerous SNPs significantly linked to disease resistance traits, as illustrated in Figure 1. However, these SNPs collectively accounted for only a modest proportion (close to zero) of the trait’s variation. Our further examination of these significant SNPs revealed no direct associations with genes of established functions. Surprisingly, SNPs linked to molecular mechanisms governing immune response and disease resistance, which one might expect, did not attain statistical significance. These findings further underscore the limitations of RAD-seq in terms of gene identification, primarily due to the provision of relatively short sequences, with an average length of only 68 base pairs.

To surmount these limitations, we are contemplating a comprehensive strategy involving the re-sequencing of a larger number of individuals to increase sequencing depth and sample size. This expanded approach aims to enhance our prospects of detecting genes that exert major effects on disease resistance traits within our studied populations of white leg shrimp, striped catfish, and yellowtail kingfish.

## 6. Genomic Prediction to Enable Genome-Based Selection

In the preceding section, we presented the outcomes of our genome-wide analysis, which reveal that numerous genetic variants additively influence disease resistance traits, with each variant exerting a relatively modest impact. Instead of searching for individual genes or variants, our approach involves simultaneously estimating their cumulative effects, leading to enhanced predictions of genetic susceptibility or genetic merit concerning disease-related phenotypes. Importantly, there are possibilities for uncovering causative mutations. Additionally, if our predictive models prove accurate, it may obviate the need for collecting phenotype data. The elimination of data recording for disease traits is particularly critical in the context of farmed and aquatic animal species, as this process entails significant time and cost implications. Furthermore, many economically significant traits are either expensive or challenging to measure, such as disease resistance, which often necessitates challenge tests involving pathogens, or eating quality traits, which require the slaughter of animals. Obviously, genomic prediction has become a pivotal component of genetic improvement programs, specifically geared towards forecasting susceptibility to infectious diseases within our studied species. The primary objective of genomic prediction is to leverage genome-wide markers or DNA sequences to forecast phenotypes, or, specifically in our studies, to predict genetic predispositions to infectious diseases, thereby facilitating genome-based selection within our target species.

In these studies, we have employed several advanced statistical methodologies to estimate genomic breeding values (gEBVs). These comprise regression techniques, Best Linear Unbiased Prediction (BLUP), and Bayesian approaches (such as Bayes A, B, C, Cpi, and R), as well as deep learning and machine learning algorithms powered by artificial intelligence [58,59]. Regardless of the method employed, the accuracy of genomic prediction is gauged by the correlation between predicted and actual phenotypes, with a correlation approaching one indicating a high level of accuracy. For instance, our predictions exhibited moderate-to-high accuracy for disease traits assessed in challenge test experiments [39], whereas accuracy was lower for disease resistance recorded under field (or farm) conditions [41]. A summary of the prediction accuracy for disease resistance traits, including survival rate and time to death, employing various statistical methods and algorithms, is shown in Table 4.

Across our studied species, a consistent trend emerged indicating that multivariate analysis increased the precision of genomic prediction for disease-related traits [39,60]. The imputation of missing genotypes also contributed to a predictive capacity enhanced by 5–18% [41]. Conversely, the utilization of SNP subsets obtained from Genome-Wide Association Studies (GWAS) yielded similar or lower prediction accuracies for these traits [39]. Generally, BLUP-based methods (e.g., GBLUP or single-step GBLUP) exhibited comparable predictive performance to common Bayesian methods (i.e., Bayes A, B, C, and Cpi). However, Bayes R marginally outperformed GBLUP and other Bayesian techniques. Both deep learning and machine learning approaches surpassed GBLUP and Bayes R to some extent; nevertheless, the advantages of AI algorithms are contingent upon the specific populations and traits under consideration [41,60]. Their benefits become more pronounced when modeling intricate interaction networks and variables (Nguyen et al., unpublished).

In summary, our findings suggest the prospects of genome-based selection to enhance disease resistance within our target populations. Also note that prediction accuracy is influenced by various factors [61], including the size of the reference population, trait heritability, structure and/or genetic composition of populations, gene or marker effects, the extent of linkage disequilibrium within populations, and the type of genotype data or sequencing platforms used (e.g., whole-genome, whole-exon, or reduced sequencing methods). In our studies, we performed reduced sequencing due to its affordable costs, which consistently provided informative markers to attain a reasonable level of prediction accuracy for disease traits. However, genomic selection for disease-related traits under field or on-farm conditions remains challenging, necessitating extensive, large-scale routine data collection pertaining to the disease status of animals during natural disease outbreaks. This indicates the continued importance of phenotype data in advancing genetic improvement for traits recorded under field (farm) environments. Furthermore, the availability of a robust genome assembly for these species holds the potential to accelerate progress in genomic selection and furnish reference information essential for comprehending the biological factors underlying genetic variations in disease traits among YTK and striped catfish populations.

## 7. Omics Technologies

With a fast advancement in high-throughput next generation sequencing platforms, we have started initial studies to study transcriptomes and metagenomes of our studied species, with the aim of integrating these new data to understand the genetics and genomic architecture of disease traits for our species.

### 7.1. Transcriptomics

Transcriptomics is the study of the transcriptome—the complete set of RNA transcripts in the genome. Our studies aimed to identify genes expressed in distinct cell populations or differentially expressed in response to various environmental factors and diseases, such as hepatopancreatic parvo-like virus (HPV). In these studies [62], we detected differentially expressed genes related to HPV in banana shrimp. Some promising candidate genes for HPV may include Kazal-type serine proteinase inhibitors (SPIs), Dicer2, and hemocyanin. In crustaceans, hemocyanin is produced by the hepatopancreas and is present in the plasma. It can be converted by hemocyte components into a phenoloxidase-like enzyme [63]. Hemocyanin has been demonstrated to function as an antimicrobial peptide and has shown non-specific antiviral properties.

Kazal-type serine proteinase inhibitors (SPIs) are promising candidates for limiting proteolytic activity in coagulation and proPO activation [64]. Recently, a Kazal-type SPI with five domains from *P. monodon* has been shown to be upregulated upon infection with WSSV, implicating it in the antiviral response [65]. These proteinases and proteinase inhibitors each display modulated expression, providing further support for their role in response to viral infection in this species.

The Dicer2 gene is involved in the RNA interference (RNAi) pathways. In high-HPV shrimp, this gene is over-expressed, suggesting that this pathway is likely upregulated in response to the higher concentration of the HPV virus. 

Despite the crucial roles of these genes, they should be validated in independent populations, and, in future studies, we will determine whether their expression patterns are inherited by offspring generations.

### 7.2. Metagenomics

Metagenomics and microbiome studies delve into the intricate world of microorganisms, exploring not only their genetic makeup, but also the environments they inhabit [66]. In our studies, we have set out to address four pivotal inquiries:

1. To what extent does genetic variation in hosts influence the microbiomes of fish and shrimp? For example, why do individuals exhibit disparities in their immune response?
2. How do host genetics interact with the microbiome to shape host phenotypes, such as susceptibility to diseases?
3. Can the microbiome serve as a reliable biomarker for predicting various phenotypes?
4. Are there specific genes or genomic regions that exert control over microbial composition?

Investigations in both humans and animals have provided evidence of host genetic variability impacting gut and ruminant microbiomes. For instance, the *Christensenellaceae* family, with a heritability estimate ranging from 0.39 to 0.62, has been associated with lean body mass index [67]. In the case of Holstein cows, Wallace et al. [68] reported significant heritable genetic variations in rumen microbial communities, suggesting the potential for breeding animals with beneficial microbiomes to enhance ruminal acetate and proportionate concentration. Beyond the scope of host genetics, both studies supported the crucial roles of environmental factors and the effects of host genetics–microbiome interaction on various phenotypes. Given the evidence from these studies, we have emphasized addressing the third question—can the microbiome serve as a reliable biomarker for predicting phenotypes? To this end, we embarked on Amplicon sequencing (or 16S ribosomal RNA sequencing) of a pedigreed population of white leg shrimp. Our preliminary findings indicate that the predictive accuracy using microbiome data is low to moderate for WSSV in this population (Nguyen et al., unpublished results). This limitation is likely attributable to the relatively small sample size employed in our study. A comprehensive review of the literature spanning over a decade reveals that metagenomic predictions hold promise for forecasting disease phenotypes (Ross and Hayes, 2022 [69]). A combined analysis of genomic and metagenomic information has enhanced the accuracy of disease phenotype predictions [70]. When more data have been accumulated, we will conduct a complete analysis to better utilize the microbiome data in genetic enhancement programs for our populations and other aquaculture species.

### 7.3. Other Omics

Other omics technologies, including proteomics, metabolomics, lipidomics, and various others [71], play a crucial role in providing molecular information regarding how genetic variants or Quantitative Trait Loci can impact gene expression at multiple levels, encompassing post-transcriptional, post-translational, or metabolomic processes. Quantitative Trait Loci (QTLs) denote genetic variants that exhibit significant associations with specific phenotypes, such as diseases. Correspondingly, eQTLs refer to genetic variants where sequence variations correlate with the altered expression of one or more genes [72]. Beyond eQTLs, genetic variants can also influence expression levels at different tiers, such as the protein level, termed protein expression QTLs, or post-translationally and metabolically, termed pQTLs and mQTLs, respectively. These QTLs are considered as molecular QTLs or molecular traits. Quantifying the correlations between variant genotypes and their expressions at these diverse levels is imperative to gain a deeper understanding of the molecular mechanisms underlying genetic disease resistance. These aspects represent key focal points for our future research, given the limited extent of our current knowledge, particularly in the context of the species under study.

## 8. Future Directions

### 8.1. Precision Agriculture Systems and Artificial Intelligence

**Figure 2:**
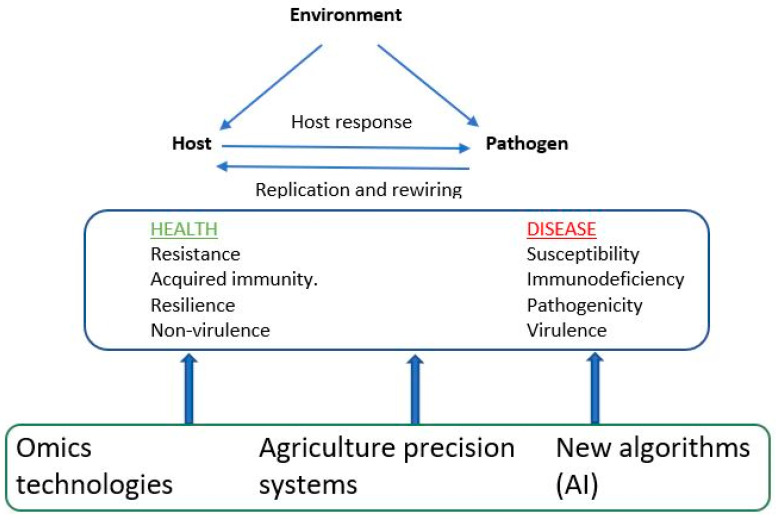
Integrated approaches to understand disease resistance.

Precision agriculture systems along with omics technologies can advance genetic improvement efforts for disease resistance traits in aquaculture species [73,74]. As illustrated in Figure 2, the genetic architecture of an infectious disease results from a complex interaction of three main components: host, pathogen, and environmental effects. Precise agriculture systems, for example, high-throughput phenotyping, which employs sensor devices and advanced imaging technologies (e.g., an underwater camera), can assist in collecting data pertaining to animal health. These data might include clinical symptoms, novel disease phenotypes, environmental factors, and disease susceptibility or resilience. Additionally, remote sensing techniques, such as satellite imagery, and the application of Geographic Information Systems (GIS) enable the analysis of spatial data related to both individual and population health, as well as disease prevalence. Furthermore, omics techniques (as discussed in Section 7 above) provide molecular information regarding the genetics of both host and pathogen effects. These technologies can integrate into automated data analytics systems that utilize AI algorithms, including machine and deep learning (Figure 2). This integration aids in the timely detection of early disease indicators, potential outbreaks, and disease-prone seasons and the identification of disease-resistant individuals. This comprehensive analysis may uncover complex relationships and provides insights into the mechanisms governing disease resistance [73]. In summary, the integration of precision agriculture systems with AI algorithms and omics technologies equips breeders with the ability to make informed, data-driven decisions concerning disease management, disease surveillance, and the selection of individuals with heightened resistance to infectious diseases among aquatic animal species. By harnessing the potential of these emerging technologies and genetic knowledge, these systems could make a substantial contribution to the development of climate- and disease-resistant animals. To date, there has been limited application of these integrated systems in practical genetic improvement programs aimed at enhancing disease resistance in aquaculture species [75]. We have started implementing these technologies to record novel disease phenotypes in our breeding programs for fish and shrimp. A comprehensive genetic/genomic analysis of these data will be reported in future studies.

### 8.2. Enhancing Overall Immune Response and Epidemiological Host Traits

Most genetic improvement programs for aquaculture species, including our studies, have attempted to develop resistant genetic strains capable of withstanding specific pathogens or diseases through challenge test experiments. However, this approach may not yield the most optimal results, as animals selected for disease resistance under controlled conditions may not thrive in real-world, on-farm environments due to the complex interplay of genotype and environmental factors. A recent study in white leg shrimp showed that the effect of G × E interactions was biologically significant as the survival and biomass production of the resistant shrimp lines differed in the presence of disease outbreaks vs. non-infected environments [76]. Furthermore, with the continuous evolution of pathogens, we devised our approaches to effectively select for disease-resistant traits. This is necessary because, in practical production systems, disease outbreaks often result from multiple pathogens, each exerting synergistic impacts on animal health. Given these challenges, a question arises: should selection strategies focus on enhancing the overall immune response of individuals within a population or on bolstering the overall health of the entire population? Some of our own research findings suggest the potential for improvement in immunological traits to improve overall health of populations [52,77]. However, future investigations should include a broader array of crucial immunological parameters, including the lysozyme assay, hemolysin test, hemagglutination test, and bacterial agglutination test, as well as cortisol, Transferrin, and Total IgM. Correlations between these traits, such as lysozyme levels, and family survival have been reported in rohu carp [56]. Importantly, immunological traits should be combined into a comprehensive immune response index. Such an index could include antibodies, cell-mediated responses, and indicators of innate immune functions. Studies in dairy cows [78] provided evidence that high-immune-response (HIR) cows exhibit desirable traits, such as reduced mastitis rates, lower incidence of infectious diseases (e.g., ketosis and retained placenta), and enhanced productivity and economic performance. Selection for HIR does not adversely impact productivity, such as milk yield. While these approaches are promising, there remains a deficiency in our understanding of immunogenetics in aquaculture species, which requires further investigations within our specific working populations. Furthermore, our recent research (Nguyen et al., in preparation) has demonstrated that conventional quantitative genetic models are inadequate for maximizing genetic gains within selected populations. Therefore, breeding objectives of genetic improvement programs for aquaculture species should be expanded to enhance epidemiological host traits, encompassing susceptibility, infectivity, and recovery [79,80]. This approach aims to fortify the overall resilience of the entire populations rather than individual animals [81,82,83]. Finally, genetic improvement strategies should be integrated with crossbreeding systems to produce animals possessing favorable attributes such as rapid growth and heightened disease resistance. These resilient traits are crucial for sustaining the aquaculture sector in the face of unpredictable climate changes, dwindling natural resources, and environmental degradation, which can contribute to reduced fitness and productivity of important aquaculture species.

### 8.3. Host–Pathogen Interactions

**Figure 3:**
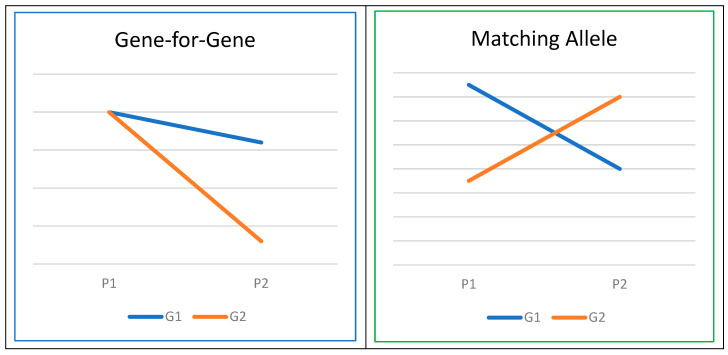
Host–pathogen interaction (G denotes genotype, and P stands for pathogen).

A foundational premise in models of host–pathogen coevolution is the existence of host genotype-by-pathogen genotype (G × G) interactions [84]. These G × G interactions can be explained through two prevailing frameworks: the ‘gene-for-gene’ (GFG) and ‘matching allele’ (MA) models of coevolution [85]. MA models propose that various host genotypes exhibit resistance or susceptibility to specific pathogen genotypes, whereas GFG models posit that host genotypes vary in their susceptibility across a range of pathogen genotypes (Figure 3). A recent review article in the field of human genetics [86] has provided evidence for G × G interactions, involving genes such as ABO, HBB, FUT2, SLC11A1, and HLA, aligning with the assumptions of either gene-for-gene or matching allele models in the coevolution of host and pathogen genomes [87]. Similar investigations have been conducted in the realm of plant biology, as documented in the review by Thrall et al. [88].

As illustrated in Figure 3, the concept of G × G interaction closely parallels that of genotype or gene-by-environment (G × E) interaction [89]. Thus, quantitative genetics theories and methodologies can be effectively leveraged to explore G × G interactions. One approach involves the integration of Genome-Wide Association Studies (GWAS) and population genomic analyses within the framework of mixed models. This combination is commonly referred to as Microbiome Genome-Wide Association Studies [90,91]. Within our target species, there is no published information, warranting further research efforts to understand effects of the G × G interactions on critical diseases. Such investigations hold the potential to optimize selection programs for combating various pathogens and diseases.

### 8.4. Genomic Surveillance in Genetic Enhancement Programs

Genomic surveillance and outbreak tracking have gained significant prominence, particularly during the COVID-19 pandemic [92]. However, there is a very limited number of publications within the field of aquaculture species, which is primarily attributable to various constraints, including limited financial and human resources, among others [7,93]. This approach leverages advanced genome sequencing techniques to analyze the genetic material of pathogens, including viruses, bacteria, and parasites, enabling the monitoring of disease spread and the understanding of their evolution (also see Figure 2). More precisely, the acquired genome sequences undergo rigorous analysis to detect genetic variations and mutations within the pathogen’s genome. These data unveil the genetic relatedness of distinct pathogen strains and illuminate their evolutionary trajectories over time. Harnessing this genomic information, we can construct phylogenetic (phylogenomic) trees to elucidate transmission chains and pinpoint infection hotspots, thereby facilitating a comprehensive understanding of outbreak dynamics—ranging from its origin and propagation to individual or population susceptibilities [94]. Insight into the genetic makeup of pathogens, including the characterization of novel variants, is also a critical component for informing the development of vaccines and treatments [95,96]. In our genetic enhancement programs for improved resistance or resilience, genomic surveillance is considered as an integral component enabling us to detect, respond to, and manage disease outbreaks in white leg shrimp, striped catfish, and yellowtail kingfish populations. Specific findings from our studies in this emerging field of research will be published in future reports.

## 9. Concluding Remarks and Suggestions

Our research has yielded valuable insights into the control of infectious diseases in white leg shrimp, striped catfish, and yellowtail kingfish. We have achieved this by developing disease-resistant strains that can thrive in diverse production systems. Despite our initial successes, it is evident that advancing genetic progress in these populations requires integrated, multidisciplinary research efforts.

In forthcoming studies, we aim to address emerging topics in the fields of genetics and genomics of infectious diseases. Exemplary questions are as follows.

Which genes or genetic mutations have the most significant impact on variations in disease severity and outcomes among host individuals?

What are the genetic determinants governing immune responses to infectious agents, and how do these responses differ among individuals?

What molecular interactions occur between highly infectious pathogens and host cells, and how does host genetics influence these interactions? Can we leverage this knowledge for genetic improvement?

What genetic mutations are responsible for changes in pathogen virulence, transmissibility, and disease dynamics?

What are the most effective strategies for integrating host and pathogen genomics to gain a comprehensive understanding of the biological factors controlling complex infectious diseases?

By addressing these questions, our future research could further enhance our ability and knowledge to combat infectious diseases in aquaculture and promote sustainable and resilient production systems.

## Figures and Tables

**Table 1:** Heritability (h^2^) for disease resistance of white shrimp (WSSV), striped catfish (BNP disease), and yellowtail kingfish (skin fluke). S.E. = standard error.

Reference	Species	n	h^2^	S.E.
Trang et al. (2019) [38]	White leg shrimp	15,000	0.130	0.028
Trang et al. (2019) [23]	White leg shrimp	120,000	0.230	0.015
Vu et al. (2019) [37]	Striped catfish	398,234	0.168	0.044
Vu et al. (2022) [39]	Striped catfish	564	0.543	0.101
Premachandra et al. (2017) [40]	Yellowtail kingfish	752	0.020	0.030
Nguyen and Vu (2022) [41]	Yellowtail kingfish	752	0.022	0.035

**Table 2:** Genetic correlations of disease-resistant traits with survival rate and growth.

Trait	Striped Catfish	White Shrimp	Yellowtail Kingfish
Survival rate	0.44 ± 0.09	−0.17 ± 0.08	n.a.
Growth	0.52 ± 0.10	0.07 ± 0.08	0.12 ± 0.27

n.a. = not available.

**Table 3:** Immune response of the high- vs. low-WSSV-resistance line in *L. vannamei* (Trang, 2020) [50].

Parameter	Unit	Line	Least Square Mean
THC	10^6^ cells·mL^−1^	High resistance	7.56 ± 0.73
		Low resistance	7.76 ± 0.92
PO	Units·mL^−1^ hemolymph	High resistance	0.035 ± 0.006
		Low resistance	0.037 ± 0.006
SOD	Units·mL^−1^ hemolymph	High resistance	0.378 ± 0.039
		Low resistance	0.354 ± 0.059
Lysozyme	Units·mL^−1^ hemolymph	High resistance	260.41 ± 4.397
		Low resistance	266.72 ± 6.388

THC = total hemocyte count, PO = phenoloxidase, SOD = superoxide dismutase.

**Table 4:** Genomic prediction accuracy for disease traits of white shrimp (WSSV), striped catfish (BNP disease), and yellowtail kingfish (skin fluke).

Method	Striped Catfish	White Shrimp *	Yellowtail Kingfish
GBLUP	0.51 ± 0.08	0.46 ± 0.06	0.23 ± 0.05
Bayes R	0.63 ± 0.09	0.73 ± 0.13	n.a.
Machine learning	0.63 ± 0.10	0.70 ± 0.11	n.a
Deep learning—MLP	0.65 ± 0.11	0.77 ± 0.15	0.17 ± 0.03
Deep learning—CNN	0.63 ± 0.12	n.a.	n.a

* banana white shrimp (*Penaeus merguiensis*). n.a. = not available.

## Data Availability

All the data are included in tables and figures of this article.
